# ZEB1/miR-200c/AGR2: A New Regulatory Loop Modulating the Epithelial-Mesenchymal Transition in Lung Adenocarcinomas

**DOI:** 10.3390/cancers12061614

**Published:** 2020-06-18

**Authors:** Lucia Sommerova, Eva Ondrouskova, Andrea Martisova, Vassilis Zoumpourlis, Sotirios Galtsidis, Roman Hrstka

**Affiliations:** 1 Research Centre for Applied Molecular Oncology, Masaryk Memorial Cancer Institute, Zluty kopec 7, 656 53 Brno, Czech Republic; luciasommerova@gmail.com (L.S.); zahradka.mail@seznam.cz (E.O.); andrea.martisova@mou.cz (A.M.); 2 Biomedical Applications Unit, Institute of Biology, Medicinal Chemistry & Biotechnology, NHRF 48 Vassileos Constantinou Ave., 11635 Athens, Greece; vzub@eie.gr; 3 Life Sciences Research Unit, University of Luxembourg, Campus Belval, Biotech 1, Avenue des Hauts Fourneaux, L-4362 Esch-sur-Alzette, Luxembourg; sotirios.galtsidis@uni.lu

**Keywords:** AGR2, EMT, ZEB1, cancer

## Abstract

Epithelial-mesenchymal transition (EMT) is a process involved not only in morphogenesis and embryonic development, but also in cancer progression, whereby tumor cells obtain a more aggressive metastatic phenotype. Anterior gradient protein 2 (AGR2) maintains the epithelial phenotype and blocks the induction of EMT, thus playing an undeniable role in tumor progression. However, the mechanism through which AGR2 expression is regulated, not only during EMT, but also in the early stages of cancer development, remains to be elucidated. In the present study, we show an inverse correlation of AGR2 with ZEB1 (zinc finger enhancer binding protein, δEF1) that was verified by analysis of several independent clinical data sets of lung adenocarcinomas. We also identified the ZEB1 binding site within the *AGR2* promoter region and confirmed *AGR2* as a novel molecular target of ZEB1. The overexpression of ZEB1 decreased the promoter activity of the *AGR2* gene, which resulted in reduced AGR2 protein level and the acquisition of a more invasive phenotype of these lung cancer cells. Conversely, silencing of *ZEB1* led not only to increased levels of AGR2 protein, but also attenuated the invasiveness of tumor cells. The *AGR2* knockout, vice versa, increased ZEB1 expression, indicating that the ZEB1/AGR2 regulatory axis may function in a double negative feedback loop. In conclusion, we revealed for the first time that ZEB1 regulates *AGR2* at the transcriptional level, while AGR2 presence contributes to *ZEB1* mRNA degradation. Thus, our data identify a new regulatory mechanism between AGR2 and ZEB1, two rivals in the EMT process, tightly associated with the development of metastasis.

## 1. Introduction

The development of metastasis represents the most crucial challenge in the treatment of cancer and remains the major cause of cancer related death. Metastatic spread of cancer represents a complex program, during which cancer cells must undergo many changes associated with increased motility, invasiveness, and loss of cell-cell junctions. This conversion is an adaptation of the normal developmental process called epithelial-mesenchymal transition (EMT). 

Although EMT term suggests the strict binary conversion from one phenotype to another, new studies showed that EMT is an extremely complex and dynamic process that gives rise to the wide spectrum of intermediate states. The intermediate or hybrid epithelial/mesenchymal phenotypes are characterized by the co-existence of both epithelial and mesenchymal traits and differ in combined expression of epithelial and mesenchymal markers reflecting the ability of the cells to acquire an EMT-associated phenotype manifested by stemness, tumorigenicity, metastatic ability, and resistance to therapy [1,2,3]. The switch in gene expression and associated phenotypic conversion is achieved by the orchestrated and coordinated action of transcription factors (TFs), which bind to their corresponding sequences, leading to the repression of epithelial markers (such as E-cadherin) and conversely to the activation of genes associated with the mesenchymal phenotype [4,5]. 

The three families of TFs SNAIL, TWIST, and ZEB occupy a privileged position in the triggering of EMT [6]. ZEB1 protein (zinc finger enhancer binding protein, δEF1), similarly to the SNAIL and TWIST family, includes two dominant members, ZEB1 and ZEB2, that regulate the expression of genes involved in the control of cell polarity and adhesiveness [7,8]. Moreover, these proteins have been described as the important players regulating various processes during an organism’s development, as well as fibrosis and cancer progression, including the regulation of metastasis by the regulation of EMT [9]. Higher levels of ZEB1 are associated with aggressive cancer features, high tumor grade, resistance to therapy, metabolic plasticity, increased incidence of metastasis, and worse clinical prognosis in the vast majority of human cancers [10,11,12]. As a transcription factor, ZEB1 binds directly to specific DNA sequences known as E-boxes (CANNTG) in the promoter region of target genes [11]. 

Anterior gradient protein 2 (AGR2) is highly conserved in vertebrates, playing an important function in the development of cement gland in *Xenopus laevis* [13]. In humans, altered expression of AGR2 was described in various adenocarcinomas, such as breast, esophagus, pancreas, lung, and ovary [14] and has been shown to contribute to the acquisition of several cancer cells hallmarks, such as tumor proliferation, anchorage-independent tumor growth, formation of metastasis, and resistance to apoptosis and chemotherapy. Although the functions of AGR2 in cancers have been studied intensively in recent years, so far only a few strategies have been demonstrated to regulate AGR2 expression, such as hormone dependent regulation [15], various microRNAs [16,17], shortening of 3’UTR (3’untranslated region) mRNA [18], and endoplasmic reticulum stress [19]. However, the regulatory mechanism responsible for the alterations in AGR2 expression during the reversible transition between the epithelial and mesenchymal phenotype still remains obscure. 

Therefore, based on recent findings demonstrating the contribution of AGR2 to the EMT and cancer progression, in addition to in silico analysis of *AGR2* promoter predicting a binding site for ZEB1 [20], we aimed to confirm regulatory effect of ZEB1 on the expression of AGR2. To minimize the impact of hormone regulation on AGR2 expression, we analyzed the ZEB1/AGR2 relationship in lung adenocarcinomas, where AGR2 expression was previously described [21,22,23]. In our present study, we demonstrate the ZEB1-mediated repression of *AGR2* and propose the existence of a negative feedback regulatory mechanism through which AGR2 controls the stability of *ZEB1* mRNA.

## 2. Results

### 2.1. Expression Profiling of AGR2 in Relation to ZEB1 in Lung Cancer Samples

**Figure 1:**
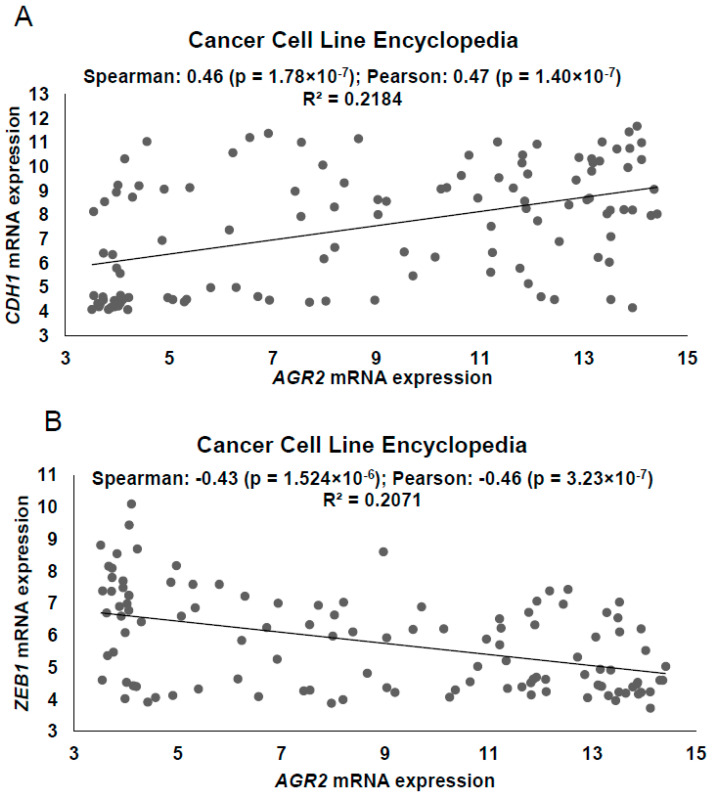
*AGR2* negatively correlates with *ZEB1* in human cancer cell lines and clinical samples. Determination of *AGR2* mRNA expression with respect to (**A**) *CDH1* and (**B**) *ZEB1* mRNA levels extracted from CBioPortal database containing the Cancer Cell Line Encyclopedia. The value of Spearman’s and Pearson’s correlation coefficient was generated by the CBioPortal database using the default set-up. Scatter plots showed a positive correlation with epithelial *CDH1* mRNA (Spearman *r* = 0.46; Pearson *r* = 0.47) and a negative correlation between *AGR2* and *ZEB1* mRNA levels (Spearman *r* = −0.43; Pearson *r* = 0.46) in cancer cell lines.

Our study emerged from three pieces of currently available knowledge about AGR2 and ZEB1: a) AGR2, similarly to all epithelial proteins, is downregulated during the EMT process [24]; b) upregulation of the EMT-associated transcription factor (EMT-TF) ZEB1 represses the expression of epithelial proteins, among which E-cadherin serves as a prominent example of ZEB1 targets [5]; and c) AGR2 protein is overexpressed in various solid malignancies including lung cancer [23,25]. However, a precise description of the related regulatory mechanism is still missing within the international bibliography. We used the CBioPortal database [26,27] to analyze the mutual relation linking *AGR2* and E-cadherin (*CDH1*) (Figure 1A) in cancer cell lines listed in the Cancer Cell Line Encyclopedia [28] (CCLE). 

The positive correlation between AGR2 and E-cadherin expression suggests that AGR2 is also an epithelial marker. Following this, we tested the hypothesis that ZEB1 could be involved in the negative regulation of AGR2, similar to ZEB1-mediated regulation of E-cadherin expression. Indeed, we revealed for the first time the existence of a strong inverse correlation between mRNA levels of *ZEB1* and *AGR2 in vitro* (Figure 1B). Moreover, we examined the expression of *AGR2* and *ZEB1* in normal lung tissues and lung adenocarcinoma tissues using the public database Oncomine [29]. Various datasets of lung adenocarcinomas (Okayama, Selamat, Landi) showed that, while average fold change of *AGR2* mRNA was significantly higher in cancer samples in comparison with corresponding normal tissues, *ZEB1* mRNA was decreased in lung adenocarcinoma specimens as compared to the normal tissues (Figure S1). To support the negative correlation between AGR2 and ZEB1 observed in Figure S1, we analyzed *AGR2* and *ZEB1* expression in two other independent databases. In Silico Transcriptomics Online-Integrated gene expression reference database (IST) confirmed both positive association between *AGR2* and *CDH1* and inverse relationships of *AGR2* with *ZEB1* (Figure S2). Similar outputs were also obtained by data mining from The Cancer Genome Atlas (TCGA) cohorts for lung adenocarcinoma [30] (Figure S3), demonstrating a significant negative correlation between *AGR2* and *ZEB1* (*p* < 0.001). 

### 2.2. Alteration in ZEB1 Expression Affects AGR2 mRNA and Protein Level

**Figure 2:**
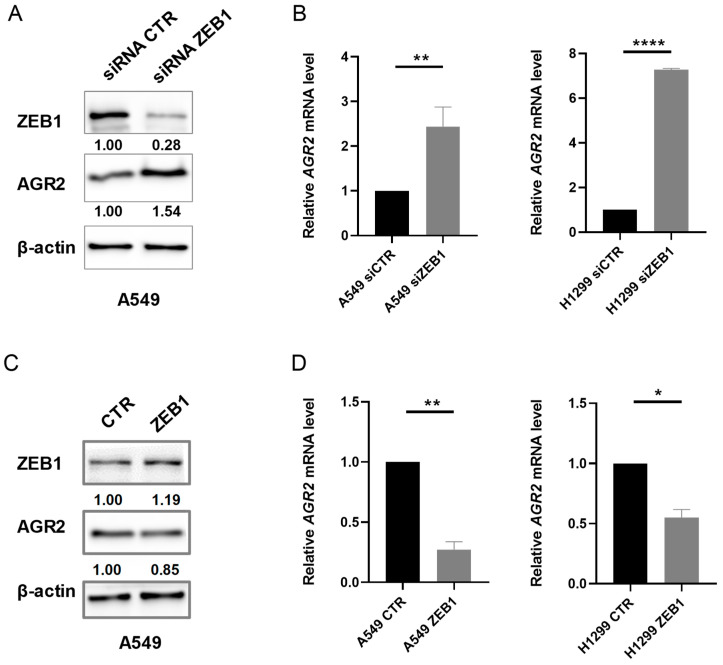
ZEB1 regulates AGR2 at mRNA and protein level. (**A**) The expression of ZEB1 was silenced with a specific siRNA (siZEB1) and the changes in AGR2 protein level were determined by Western blot analysis. β-actin served as a loading control. (**B**) Quantitative analysis of *AGR2* mRNA was performed in A549 and H1299 cells transfected with specific siRNAs against *ZEB1* or control siRNA (siCTR)*. 18S* rRNA (ribosomal RNA) served as an endogenous control for data normalization. (**C**) Western blot analysis in A549 cells transfected with plasmid coding for *ZEB1* or control plasmid (CTR). (**D**) A549 and H1299 cells were transfected with plasmid coding for *ZEB1* to determine the effect of enhanced ZEB1 expression on *AGR2* mRNA levels by Real-Time PCR (polymerase chain reaction). *18S* rRNA served as an endogenous control for data normalization. Data plotted into the graphs are the mean ± standard deviation (SD) obtained from three independent experiments. * *p* ≤ 0.05, ** *p* ≤ 0.01, **** *p* ≤ 0.0001. Full Western Blots are shown on Figure S5.

The inverse correlation of *ZEB1* mRNA with *AGR2* mRNA in lung tumor samples suggests the possibility that ZEB1 may negatively regulate AGR2 expression. Therefore, we selected lung cancer cell lines A549 and H1299 differing in AGR2 expression to study in more detail the crosstalk between ZEB1 and AGR2 proteins. Transient transfection with *ZEB1* specific small interfering RNA (siRNAs) resulted into approximately 50% increase of AGR2 protein level in A549 cells (Figure 2A). The negative effect of ZEB1 on AGR2 was also confirmed at the transcriptome level, since *ZEB1* silencing induced *AGR2* mRNA in both A549 and H1299 cells (Figure 2B). Conversely, the transfection with plasmid coding full-length *ZEB1* led to a moderate decrease in AGR2 protein level (Figure 2C) and significant decrease in *AGR2* mRNA levels (Figure 2D). These data indicate that ZEB1 suppresses the transcription of *AGR2*, which is also reflected in AGR2 protein levels.

### 2.3. ZEB1 Interacts with AGR2 Promoter to Repress Gene Expression

**Figure 3:**
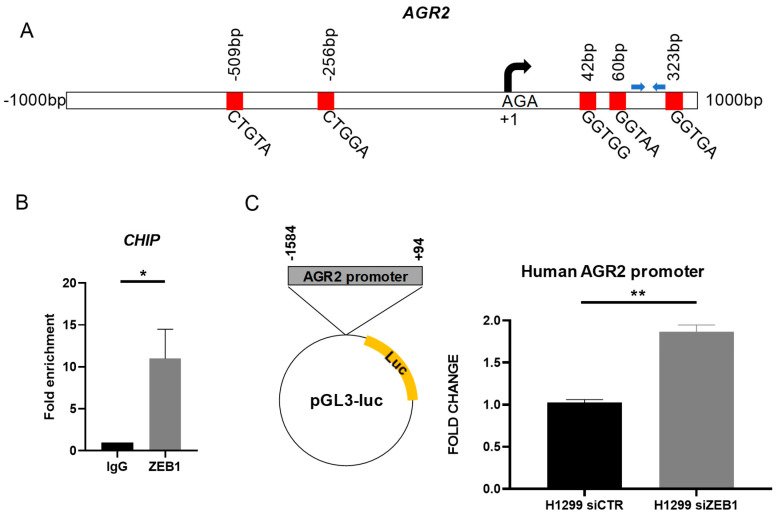
ZEB1 directly binds to the promotor of the *AGR2* gene to repress its expression under basal conditions. (**A**) Schematic representation of potential E-boxes located in the *AGR2* promoter. Blue arrows indicate primer binding sites used for the chromatin immunoprecipitation (ChIP) assay. (**B**) ZEB1 associates with the *AGR2* promoter in A549 cells as shown by ChIP analysis. Input DNA samples were used for normalization and rabbit ZEB1 antibody or control immunoglobulins (IgG) were used for ChIP experiments. * *p* ≤ 0.05. (**C**) AGR2 negative cells H1299 were transfected with specific siRNA against *ZEB1* (siZEB1) or control siRNA (siCTR) for 24 hours and with plasmid coding for *AGR2* promoter sequence from −1584 to +94 or with control pGL3-luc plasmid for additional 24 hours and the luciferase signal was determined by a spectrophotometer, whereas a Renilla luciferase empty plasmid was used for normalization of the transfection efficiency. Data plotted into the graphs are the mean ± standard deviation (SD) obtained from three independent experiments. ** *p* ≤ 0.01.

In order to describe the molecular mechanism by which ZEB1 inhibits *AGR2* expression in more detail, we analyzed in silico the presence of ZEB1 specific response elements, called E-boxes, within the region of −1 kb upstream to +1 kb downstream of *AGR2* transcription start site. Using the JASPAR database [31] and the Eukaryotic Promoter Database (EPD) [32], five putative ZEB1 binding sites were identified (positions: −509/−504, −256/−251, 42/47, 60/65, 323/328) in above mentioned region (Figure 3A). The presence of ZEB1-*AGR2* promoter DNA interaction was subsequently confirmed by chromatin immunoprecipitation (ChIP), followed by qPCR analysis (Figure 3B). Following this, luciferase reporter assay was used to confirm that binding of ZEB1 to the *AGR2* promoter represses the transcription of *AGR2*. Briefly, H1299 cells showing high ZEB1 level but no endogenous AGR2 expression were transfected with *ZEB1* specific siRNA and after 24 hours, the cells were co-transfected with the pGL3-luc plasmid bearing the *AGR2* promoter sequence –1584/+94 prior to the sequences coding for the *firefly luciferase* reporter gene [33]. Compared to transfection with control vector, the silencing of *ZEB1* caused a significant increase in the *AGR2* promoter activity (Figure 3C). Taken together, our data demonstrate that ZEB1 has a prominent role in the regulation of *AGR2* expression. Endogenous ZEB1 directly interacts with the E-boxes localized at the *AGR2* promoter, which results in a significantly decreased transcription of *AGR2* gene.

### 2.4. The Presence of AGR2 Regulates the Expression of ZEB1

**Figure 4:**
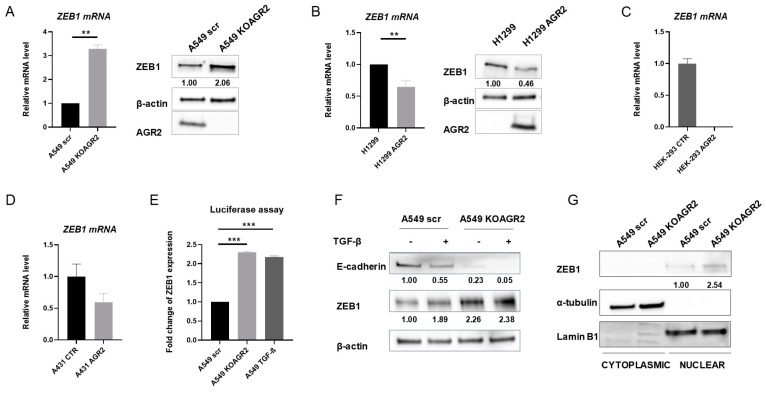
The knockout of AGR2 alters the mRNA expression of *ZEB1.* Comparison of *ZEB1* mRNA (left) and ZEB1 protein levels (right) in (**A**) A549 scr and A549 KOAGR2 cells, (**B**) H1299 and H1299 AGR2 cells, (**C**) HEK-293 CTR and HEK-293 AGR2 cells, (**D**) A431 CTR and A431 AGR2 cells. The data were normalized using *18S* rRNA for mRNA and β-actin for protein expression. The mRNA levels of *ZEB1* were determined relative to its mRNA level in A549 scr cells. The results represent the mean ± SD of at least two independent experiments (**A**,**B**) and one experiment (**C**,**D**), respectively performed in technical triplicates; ** *p* ≤ 0.01, *** *p* ≤ 0.001. (**E**) Cells were transfected with Renilla luciferase reporter plasmid pLuc-CDS coding full-length *ZEB1* to analyze the relative amount of ZEB1 in A549 scr and KOAGR2 cells either treated or untreated with TGF-β. (**F**) Immunochemical analysis of ZEB1 protein levels in cells either untreated or treated with TGF-β. (**G**) The subcellular fractionation and immunochemical analysis of ZEB1 protein in nuclear and cytoplasmic fraction. α-tubulin was used as a marker of cytoplasmic fraction and Lamin B1 was used as a marker for nuclear fraction. Full Western Blots of (**A**,**B**) are shown on Figure S6. Full Western Blots of (**F**,**G**) are shown on Figure S7.

Our previous work showed that not only AGR2 is downregulated by activation of the EMT process but also *AGR2* gene knockout mimics EMT induction as demonstrated by the acquisition of spindle-like morphology and switching of gene expression, including the increased ZEB1 level [24]. Thus, we presumed that AGR2 expression mediates the switch between the epithelial and mesenchymal phenotype through altering ZEB1 levels. To explore whether AGR2 actively regulates ZEB1, we altered AGR2 expression in A549 cells and analyzed the changes in the mRNA and protein level of ZEB1. RT-qPCR (reverse transcription-quantitative PCR) and Western blot analysis revealed that knockout of *AGR2* increased ZEB1 in both mRNA and protein levels (Figure 4A). Accordingly, *ZEB1* mRNA levels were repressed by the overexpression of AGR2 in H1299 cells (Figure 4B, left part). The changes observed in the mRNA level were also reflected in the protein level as demonstrated by Western blot analysis (Figure 4B, right part). To exclude the possibility that mutation in *RAS* oncogene family members may contribute to the negative correlation between AGR2 and ZEB1, we also analyzed the *ZEB1* level with respect to AGR2 expression in two different cell lines, HEK-293 and A431, with wild-type *RAS* (Figure 4C,D). 

Since the expression of both AGR2 and ZEB1 is regulated during TGF-β induced EMT, we compared the effect of *AGR2*-knockout and TGF-β treatment on ZEB1 activity using a luciferase reporter assay. Interestingly, loss of AGR2 and TGF-β treatment induced ZEB1 to a similar extent (Figure 4E). In agreement with this data, we observed the increased ZEB1 protein level in response to both TGF-β treatment and *AGR2* gene knockout (Figure 4F). However, no significant additive effect on the ZEB1 protein level was observed after combining *AGR2* knockout with TGF-β treatment. Correct subcellular localization of specific transcription factors represents another important prerequisite for efficient transcription. Thus, the potential involvement of AGR2 in the regulation of ZEB1 subcellular distribution was also analyzed. Immunochemical analysis of nuclear and cytoplasmic fractions revealed that *AGR2* knockout resulted predominantly in increased accumulation of ZEB1 in the nucleus (Figure 4G), indicating that AGR2 presence may also attenuate the transcriptional activity of ZEB1 by regulating its nuclear level.

### 2.5. AGR2 Decreases the Stability of ZEB1 mRNA

**Figure 5:**
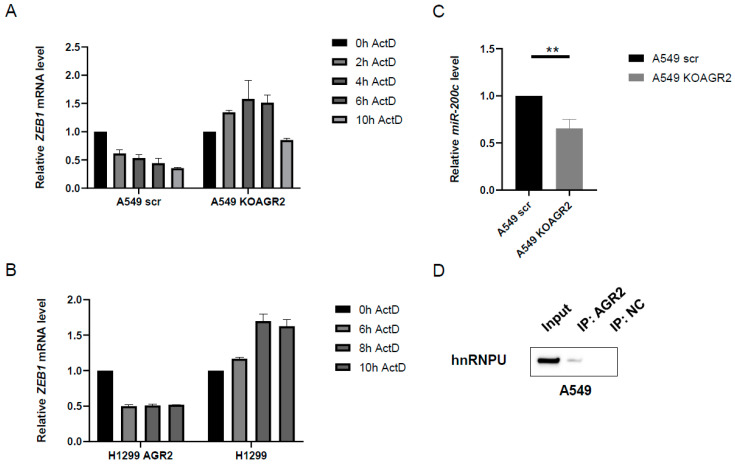
AGR2 regulates the stability of the *ZEB1* mRNA level. Analysis of the *ZEB1* mRNA level in (**A**) A549 scr and A549 KOAGR2 cells and (**B**) H1299 and H1299 AGR2 cells exposed to actinomycin D as indicated. (**C**) RT-qPCR analysis of the miR-200c level in A549 scr and A549 KOAGR2 cells. *RNU48* was used as housekeeping control for microRNA (miRNA) levels. ** *p* ≤ 0.01 (**D**) Protein-protein immunoprecipitation with a specific antibody recognizing AGR2, followed by immunochemical analysis of hnRNPU protein in A549 cells. A non-specific antibody was used as a negative control (NC) for the immunoprecipitation experiments. Full Western Blots are shown on Figure S8.

Since AGR2 is a protein disulfide isomerase without a described role as a transcriptional factor, and the alteration of AGR2 expression is reflected into *ZEB1* mRNA changes and nuclear protein level of ZEB1, we focused on the mechanism used by AGR2 to modulate ZEB1 expression, especially at the post-transcriptional level, by regulating the stability of *ZEB1* mRNA. We analyzed the changes in the *ZEB1* mRNA level under conditions during which cells showing different AGR2 expression were treated with the transcription inhibitor actinomycin D for varying time periods. Both AGR2-positive cell lines (A549 scr and H1299 AGR2) showed a significantly faster decrease of *ZEB1* mRNA in response to actinomycin D treatment, as compared to their AGR2-negative counterparts (Figure 5A,B). The half-life of *ZEB1* mRNA in AGR2 positive cells was about 6 hours, in contrast to AGR2 negative cells, which showed a *ZEB1* mRNA half-life greater than 10 hours under exposure to actinomycin D. These findings suggest that the presence of AGR2 significantly influences *ZEB1* mRNA susceptibility to degradation.

Potential mechanism would be linked to the presence of miR-200c, a potent negative ZEB1 regulator, which may, in cooperation with AGR2, decrease *ZEB1* mRNA stability and level in general [12,34]. Therefore, we analyzed the miR-200c level in both AGR2 positive (scr) and *AGR2* knockout cells (KOAGR2). Interestingly, miR-200c was significantly (*p* = 0.0032) downregulated in A549 KOAGR2 cells in contrast to cells expressing AGR2 (Figure 5C). This finding supports the hypothesis that in cells lacking AGR2, *ZEB1* mRNA is more stable due to the lower level of miR-200c. Moreover, these results reveal for the first time the novel function of AGR2 as an important regulator of mRNA stability. Analysis of AGR2 interactome (our yet unpublished data) revealed the interaction of AGR2 with several RNA-binding proteins, indicating that AGR2 may constitute part of larger multiprotein complexes and either directly or indirectly contributes to mRNA processing. This hypothesis is supported by the immunoprecipitation experiment, which confirms the interaction of AGR2 with hnRNPU (heterogeneous nuclear ribonucleoprotein U), one of the nucleic acid binding proteins (Figure 5D) previously described as an activator of the miR-200 family [35]. hnRNPU in complex with histone acetyltransferase PCAF counteracts the ZEB1 suppressive effect on the *miR-200c* promoter, which leads to the increased expression of miR-200c [36,37]. Collectively, these data unveil the existence of a new regulatory multiprotein complex associated with miR-200c, which plays an important role in the regulation of *ZEB1* mRNA levels.

### 2.6. AGR2 Knockout Enhances Aggressive Phenotype through ZEB1

Our data mining in public databases revealed a higher AGR2 expression in lung adenocarcinomas as compared to normal lung tissues (Figure S1), which may instantly suggest that AGR2 plays an oncogenic role in lung carcinogenesis. However, comparison of AGR2 expression between the primary and metastatic tumors in general showed lower levels of AGR2 in the metastatic tumors with respect to the primary tumor site (Figure S4). Therefore, looking from a different point of view, although the differences are statistically not significant (*p* = 0.7635 for Bittner and *p* = 0.2824 for Bhattacharjee dataset), presumably due to the low number of available metastatic samples, we observe a remarkable trend indicating that the absence of AGR2 could be associated with an increased rate of metastasis formation. This phenomenon is also supported by the inverse correlation between AGR2 and ZEB1, since ZEB1 is strongly upregulated in invasive and metastatic lung cancer cells, but its expression is downregulated in the early phases of lung carcinogenesis [38].

**Figure 6:**
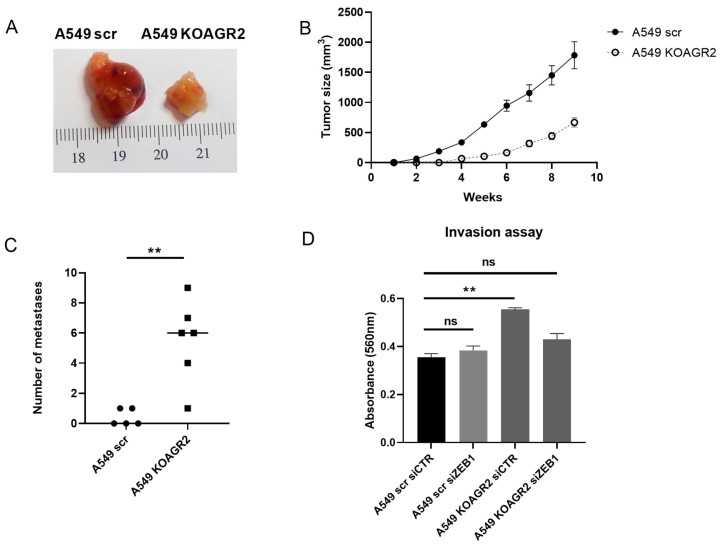
The alteration in AGR2 expression regulates an aggressive phenotype in vitro and in vivo. (**A**) Illustration of primary tumors developed in mouse xenografts. A549 scr (*n* = 5) and A549 KOAGR2 (*n* = 6) cells were injected into the flanks of mice with severe combined immunodeficiency (SCID). (**B**) Growth rate of tumors developed from A549 scr and A549 KOAGR2 cells. (**C**) The graph showing the frequency of metastasis development derived from Table S1. (**D**) An invasion assay was performed to analyze the number of cells that invaded through a semi permeable membrane. Invaded cells were stained and measured by spectrophotometer. The results of invasion experiments are an average of 3 technical replicates from 2 independent experiments, plotted as a mean ± SD where ** is ** *p* ≤ 0.01 and “ns” means statistically non-significant (*p*> 0.05).

To get a deeper insight on the role of AGR2 in metastasis, the effect of *AGR2* knockout with respect to tumor growth and metastasis formation was analyzed using a mouse xenograft model. We observed that mice subcutaneously injected with control A549 scr cells developed visible tumors within 2 weeks post-injection and that tumors grew to a size of up to 1784 mm^3^ within 9 weeks. In contrast, mice injected with A549 KOAGR2 cells showed delayed tumor formation, with the first visible tumor observable after 4 weeks, and the size of these slow-growing tumors reached only up to 668 mm^3^ after 9 weeks, when the experiment was terminated (Figure 6A,B).

On the other hand, we found that in mice injected with parental A549 scr cells, only two out of five developed at least one lung metastasis lesion, while in the group of mice injected with A549 KOAGR2 cells, all mice showed development of lung metastasis lesions (Figure 6C, Table S1). Moreover, 83% of mice with *AGR2* knockout cells showed more than four micrometastatic lesions. 

While these findings support the functional role of AGR2 as a positive regulator of tumor growth, they also show that AGR2 depletion may promote the escape of tumor cells from the site of injection and their spread to the lungs. To evaluate whether knockout of *AGR2* contributes to the acquisition of a more aggressive phenotype due to increased levels of ZEB1, we silenced *ZEB1* expression with a specific siRNA. Indeed, we found that inhibition of ZEB1 counteracted the effects of *AGR2* knockout on the invasiveness of tumor cells, as shown by the invasion assay. Cells with *AGR2* knockout exhibited higher invasiveness as compared to cells expressing AGR2; however, *ZEB1* silencing significantly reduced the invasion rate of these cells (Figure 6D). Based on these data, we can conclude that alteration of the balance between AGR2 and ZEB1 in favor of ZEB1 leads to the acquisition of an aggressive and more invasive phenotype of tumor cells. 

## 3. Discussion

Since the presence of metastasis remains the most common cause of cancer-related death, identification of genes associated with metastasis development and characterization of their regulation mechanism may lead to the development of new, more effective therapies targeting metastatic disease. Epithelial-mesenchymal transition and its reverse process, mesenchymal-epithelial transition (MET), play an important role in embryogenesis, organ fibrosis, stem cell biology, and cancer progression [39]. These processes are regulated by coordinated changes in the expression of core transcription factors ZEB1/2 SNAI1/2, TWIST1/2, acting as repressors of the epithelial phenotype. Although increased efforts are devoted to understanding the machinery of EMT and MET, many regulators of these processes, which represent potential therapeutic targets, still remain elusive. Previous reports have shown that both AGR2 and ZEB1 are involved in the regulation of EMT/MET [10,12,24,40], processes closely related to metastasis promotion [41]; however, the mutual relation between AGR2 and ZEB1 had not been suggested so far. This constitutes the reason why we aimed to investigate the relationship between AGR2 and ZEB1 and how their crosstalk could contribute to the process of EMT. Epidermal growth factor receptor (EGFR) mutations are one of the key characteristics of lung adenocarcinomas and their presence predicts treatment decision [42,43]. Interestingly, the study by Zhang et al. showed that EGFR mutations positively correlate with the loss of ZEB1 [38]. Furthermore, increased expression of AGR2 could serve as a positive biomarker of mutated EGFR predicting the sensitivity to anti-EGFR therapy [44]. When linked together, these studies present yet another suggestion for the inverse relationship between ZEB1 and AGR2. In the present work, we demonstrate for the first time that AGR2 and ZEB1 expression shows an inverse correlation in lung adenocarcinomas, since AGR2 is usually overexpressed in tumor tissue as compared to the normal tissues, while ZEB1 shows the opposite trend. However, in the late and metastatic stages of lung cancer, expression profiles are changed in favor of ZEB1 [12,45], which is associated with a decreased level of AGR2 (Figure S4). 

Although the involvement and molecular function of AGR2 in tumorigenesis has been exponentially reported over the past decade, its regulation during cancer development and progression has not been completely elucidated. In addition to stress conditions such as hypoxia and ER stress that regulate AGR2 protein expression, several transcription factors have been described to be involved in *AGR2* promoter activation, predominantly FOXA1/2, FOXM1, and recently TWIST1 [22,46,47]. Similar to ZEB1, TWIST1 belongs to the EMT-associated TFs, but the effect on AGR2 expression remains unclear, as the only two articles published so far present contradictory results on TWIST1 and AGR2 [46,48]. In contrast, our data clearly demonstrate that AGR2 expression is suppressed by ZEB1 expression in lung cancer cells. At the same time, these results do not exclude that the various TFs associated with EMT may influence AGR2 in various manners and to a different extent. 

Interestingly, we confirmed the existence of a double-sided regulation that involves AGR2, miR-200c, and ZEB1. The ChIP assay confirmed the previous prediction that ZEB1 binds to the *AGR2* promoter and represses the transcription of *AGR2* [20]. Similarly, ZEB1 binds to the promoter of *miR-200c* and represses transcription of this miRNA [49]. However, miR-200c is also one of the essential repressors of ZEB1 in cancer cells, since it binds directly to the 3’UTR region of *ZEB1* mRNA and enhances its degradation [34,50]. Complementary to these findings, we show that AGR2 protein significantly contributes to miR-200c-dependent suppression of ZEB1, and in this way, AGR2 together with *miR-200c* prevent the acquisition of an aggressive phenotype, since decreased *ZEB1* expression is tightly associated with the attenuation of migratory and invasive properties of cancer cells [51,52]. In line with these findings, we also show that knockout of *AGR2* not only upregulates ZEB1 expression and activity (Figure 4A–C), but it also enhances the metastatic dissemination of lung cancer cells (Figure 6C). An observed mutual relationship between AGR2/miR-200c is supported by Ljepoja et al., showing that knockout of miR-200c leads to significant downregulation of AGR2, which is associated with advanced cancer-subtypes due to activated EMT, which is in turn associated with increased migration and chemoresistance [53]. Taken together, these data indicate that the expression of AGR2 may be attenuated in tumor cells directly by binding ZEB1 to the *AGR2* promoter or indirectly by inhibition of *miR-200c* transcription. 

Our data demonstrate that the alterations in AGR2 expression affect the stability of *ZEB1* mRNA, since *ZEB1* mRNA is more stable in cells lacking AGR2. In contrast, the presence of AGR2 significantly enhances the degradation of *ZEB1* mRNA. These findings are also supported by the protein-protein interaction of AGR2 with hnRNPU, which is part of the complex consisting of actin and histone acetyltransferase p300/CBP-associated factor (PCAF) [37]. Interestingly, this ribonucleoprotein complex predominantly responsible for the regulation of RNA polymerase II transcription elongation was previously shown to be involved in the regulation of the miR-200 family [35].

These findings, together with our in vivo data, offer new insight into cancer progression and metastatic cascade. We show that lung cancer cells A549 with endogenous expression of AGR2, when injected subcutaneously, form more easily primary tumors compared to A549 cells with *AGR2* gene knockout which generate significantly smaller tumors. This is in accordance with the previously described function of AGR2 as the inducer of tumor growth [22]. Moreover, Milewski et al. also showed that *AGR2* is transcriptionally activated by FOXM1, resulting in mucinous character, accelerated growth, and invasiveness of these adenocarcinomas [22]. However, our results rather indicate that presence of AGR2 inhibits EMT, whereas A549 cells with silenced *AGR2* are more prone to develop lung micrometastases in mouse xenografts in contrast to cells with endogenous AGR2 overexpression. Therefore, we hypothesize a model in which regulated modulation of AGR2 expression may serve as a driver of metastatic progression. Higher expression of AGR2 in a primary site may serve as the growth promoter, while activation of EMT program decreases AGR2 level, thus helping cancer cells to disseminate, and finally, MET switch may re-activate expression of AGR2, leading to enhanced adhesion and easier colonization of a secondary site. 

## 4. Materials and Methods

### 4.1. Cell Lines and Reagents

Lung cancer cell lines: A549 (ATCC^®^ CCL-185™) and H1299 (ATCC^®^ CRL-5803™) as well as the epidermoid carcinoma derived A431 (ATCC^®^ CRL-1555™) and immortalized HEK 293 (ATCC^®^ CRL-1573™) derived from human embryonic renal epithelium, were maintained in high glucose Dulbecco’s Modified Eagle’s Medium (DMEM, Sigma-Aldrich, St. Louis, MO, USA) supplemented with 10% fetal bovine serum (FBS, Life Technologies, Darmstadt, Germany), 1% pyruvate, and L-glutamine at 37 °C in a humidified atmosphere of 5% CO_2_. All cell lines were obtained from American Type Culture Collection (ATCC, Manassas, VA, USA). Unless otherwise stated, cells were grown to 70–80% confluence prior to treatment. TGF-β (R&D Systems, Minneapolis, MN, USA) was added to a final concentration of 1 ng/mL for 24 hours and 10 µg/mL actinomycin D (Life Technologies, Darmstadt, Germany).

Cells were transfected with 2 μg of plasmid DNA or 50 pmol of siRNA oligonucleotides (Dharmacon, ThermoFisher Scientific, Pittsburgh, PA, USA) per million cells. The Flp-In^TM^ System (Invitrogen, Carlsbad, CA, USA) was used to generate H1299-LZ4 cells (hereinafter H1299) containing a single integrated Flp Recombination Target (FRT) site. The coding sequence of the human *AGR2* gene was stably inserted into this site using Flp recombinase mediated site-specific DNA recombination to give H1299-LZ4-AGR2 (H1299 AGR2) cells. Cell lines with *AGR2* gene knockout were prepared as described previously using CRISPR/Cas9 [24]. Briefly, A549 cells were transfected with plasmid LentiCRISPR-v2_AGR2 or LentiCRISPR-v2_scrambled serving as a control. Then, the cells were exposed to puromycin for several weeks. Clones were selected from the pool of resistant cells and tested for AGR2 expression and validated by sequencing. Two cells clones were further used: A549 scrambled (A549 scr, with AGR2 expression) and A549 KOAGR2 (without AGR2 expression). 

### 4.2. Gene Expression

Total RNA was isolated using Ribozol reagent (VWR, Lutterworth, UK). The cDNA was synthesized by RevertAid H Minus Reverse Transcriptase (Life Technologies, Darmstadt, Germany). Either SYBR Green MasterMix (Roche, Basel, Switzerland) or TaqMan Universal PCR MasterMix (Life Technologies, Darmstadt, Germany) were used for quantitative PCR. *18S* rRNA and *GAPDH* served as parallel endogenous controls. The data represent means of three technical triplicates within each independent biological replicate (*n* = 3). The primer sequences are listed in supplementary Table S2. The relative mRNA expression levels of each gene were calculated using the 2^−ΔΔCT^ method. 

### 4.3. TaqMAN Advanced microRNA Assay

MicroRNA expression levels were determined using TaqMan Advanced miRNA Assays (ThermoFisher Scientific, Waltham, MA, USA) according to the manufacturer’s protocol. The final data show means of three technical triplicates within each biological replicate (*n* = 3) and the obtained average C_T_ values for target miRNA and RNU48 serving as a reference for data normalization were used to calculate relative gene expression using the 2^−ΔΔCT^ method.

### 4.4. Western Blot Analysis

Cells were washed twice with cold phosphate-buffered saline (PBS) and then scraped into NET lysis buffer (150 mM NaCl, 1% NP-40, 50 mM Tris-HCl, pH 8.0, 50 mM NaF, 5 mM EDTA, pH 8.0) supplemented with protease and phosphatase inhibitor cocktails according to the manufacturer’s instructions (Sigma-Aldrich, St. Louis, MO, USA). Following sodium dodecyl sulfate polyacrylamide gel electrophoresis (SDS-PAGE), the samples were transferred on to nitrocellulose membranes and incubated overnight at 4 °C with the primary antibodies. The following day, membranes were washed and probed with horseradish peroxidase conjugated with secondary antibodies (1:1000) for 1 hour at room temperature. Chemiluminescent signals were developed using the ECL solution and visualized with GeneTools (Syngene, Cambridge, UK). Either α-tubulin or β-actin was used as a loading control and as a reference for protein expression normalization. Subcellular fractionation using NE-PER Nuclear and Cytoplasmic Extraction Reagent (ThermoFisher Scientific, Waltham, MA, USA) was conducted according to the manufacturer’s instructions.

Antibodies: ZEB1 (Cell Signalling Technology Danvers, MA, USA, Santa Cruz Biotechnology, Dallas, TX, USA); AGR2 (K-31, in-house); Lamin B1, β-actin (Santa Cruz Biotechnology, Dallas, TX, USA), Alexa Fluor 488 goat anti-mouse IgG, Alexa Fluor 532 goat anti-rabbit IgG (both Abcam, Cambridge, UK); horseradish peroxiadase (HRP)-conjugated swine anti-rabbit and HRP-conjugated rabbit anti-mouse (both Dako, Glostrup, Denmark).

### 4.5. Luciferase Reporter Gene Assay

Cells were grown in 12-well plates and transfected using PEI (polyethylenimine, Sigma-Aldrich, St. Louis, MO, USA). Renilla luciferase reporter plasmid pLuc-CDS (#42100, Addgene, Watertown, MA, USA) bearing *ZEB1* coding region was co-transfected with mammalian reporter vector pGL3, allowing for weak constitutive expression of firefly luciferase. Twelve hours later, the cells were treated with TGF-β and luciferase activity was measured using the Dual-Luciferase Assay System (Promega Corporation, Madison, WI, USA) after an additional 24 hours incubation. The pGL3 reporter plasmid with cloned *AGR2* promoter sequence from −1584 to +96 was used to analyze the efficiency of *AGR2* transcription. In this case, Renilla luciferase vector served as an internal control. 

### 4.6. Cell Invasion Assay

Cell invasion was assessed using a CytoSelect 24-well Cell Invasion Assay kit (Cell Biolabs, San Diego, CA, USA) according to the manufacturer’s protocol. Briefly, at 36 hours post-transfection, 1 × 10^5^ cells in 300 μL serum-free medium were added to the upper chamber precoated with basement membrane matrix solution. Subsequently, 0.5 mL of 10% FBS-containing medium were added to the lower chamber as a chemoattractant. The cells were incubated for 36 hours at 37 °C, then the non-invading cells were removed with cotton swabs. The cells, which migrated to the bottom of the membrane, were fixed and stained with staining solution for 10 minutes. The inserts with stained cells were air-dried, then incubated in the extraction solution for 10 minutes and absorbance was measured at a TECAN spectrophotometer (Tecan, Zürich, Switzerland) at 560 nm.

### 4.7. Chromatin Immunoprecipitation (ChIP) Assay

A549 cells were grown to 80% confluency, collected and cross-linked for 10 minutes at 37 °C with 1% formaldehyde. Glycine was added to a final concentration of 0.5 M for 5 minutes at 37 °C. Cells were then washed with PBS, scraped and centrifuged (2400 rpm, 4 °C for 10 minutes). The supernatant was removed and the pellet was resuspended in lysis buffer (50 mM Tris-HCl, pH 7.5, 150 mM KCl, 0.1% SDS, 5 mM EDTA, 1% NP-40, 0.5% sodium deoxycholate, and 1% protease inhibitor cocktail from Sigma-Aldrich, St. Louis, MO, USA), left on ice for 10 minutes and snap frozen in liquid nitrogen. Samples were then sonicated at 4 °C (10 × 20 seconds with a 30 seconds pause, VibraCell, Sonics & Materials, Newtown, CT, USA). Supernatants were recovered by centrifugation (13000 rpm, 4 °C for 10 minutes) and precleared for 1 hour at 4 °C with protein G-sepharose beads. Beads were prepared by several wash cycles, followed by 1 hour incubation with salmon sperm at room temperature and then diluted in dilution buffer (45 mM Tris-HCl, pH 7.5, 135 mM KCl, 0.9% NP-40). Immunoprecipitations were performed overnight at 4 °C with ZEB1 (ZEB H-102, Santa Cruz Biotechnology, Dallas, TX, USA) or control IgG. The precipitated samples were washed sequentially 5 times for 10 minutes each at 4 °C in Wash Buffer (50 mM Tris-HCl, pH 7.5, 150 mM KCl, 1%NP-40, 0.25% sodium deoxycholate). Beads were eluted with 100 μL of Elution Buffer (0.1% SDS, 50 mM Tris-HCl, pH 7.5, 5 mM EDTA, 10 mM DTT). Cross-links were reversed by overnight incubation at 65°C, before DNA purification with the PCR cleanup kit (QIAGEN, Hilden, Germany) and qRT-PCR. The sequences of primers for PCR analysis are listed in Table S1.

### 4.8. Immunoprecipitation (IP)

For immunoprecipitation experiments, cells were extracted using lysis buffer supplemented with complete protease inhibitor cocktail. Cell lysates containing 200 µg of whole proteins were incubated with 1 µg/mL anti-AGR2 mouse antibody (Abnova, Heidelberg, Germany) overnight at 4 °C with gentle agitation. Immune complexes were isolated by incubation with Protein G Sepharose 4 Fast Flow beads (GE Healthcare, Chicago, Illinois, USA) at 4 °C for 2 hours, followed by five washes in lysis buffer. Immune complexes were eluted with 2× Laemmli buffer (Invitrogen, Carlsbad, CA, USA), boiled and set aside for immunoblotting.

### 4.9. Tumor Xenografts

First, 4 × 10^6^ of either A549 scr or A549 KOAGR2 cells were resuspended in 100 µL PBS and injected subcutaneously into the left and right flanks of 5–6 week old female SCID mice. Mice were divided into two groups of 6, injected with either A549 scr or A549 KOAGR2 cells. Tumors were allowed to grow for 9 weeks after the injections. During this period, tumor volumes were measured (as soon as tumor onset was observed) using a calliper and calculated using the formula ½ × height × width × length. At the end of the observation period, mice were sacrificed and tumors were excised and photographed. Primary tumors and lungs were removed, fixed, and embedded in paraffin. Hematoxylin-eosin staining was used for histopathological evaluation.

All experiments with mice were performed in the authorized animal house of the National Hellenic Research Foundation. Experiments complied with the Protocol on the Protection and Welfare of Animals, as obliged by the rules of the National Hellenic Research Foundation, the regulations of the National Bioethics Committee, and article 3 of the presidential decree 160/1991 (in line with 86/609/EEC directive) regarding the protection of experimental animals.

### 4.10. Statistical Analysis

The error bars represent the standard deviation of corresponding data sets. One-way ANOVA (analysis of variance) with post-hoc Tukey HSD (Honestly Significant Difference) calculator was used to determine statistically significant differences between the groups generated from at least three independent experiments. Statistical analysis was performed using the free online web tool denoted as One-way ANOVA (ANalysis Of VAriance) with post-hoc Tukey HSD (Honestly Significant Difference) Test Calculator for comparing multiple treatments. Tests with *p* < 0.05 were considered as significant.

## 5. Conclusions

Our data confirm the existence of the negative feedback mechanism between AGR2 and ZEB1. Moreover, the negative correlation between these two proteins seems to influence the aggressiveness of cancer cells and their metastatic dissemination. Thus, identifying the AGR2/ miR-200c /ZEB1 axis and its involvement in cancer progression could represent a new strategy leading to more efficient targeting and/or preventing the development of metastasis. Moreover, the potential role of AGR2 in the assembly of RNA-binding protein complexes represents a new direction for additional research focusing on AGR2 as the regulator of gene expression at the post-transcriptional level.

## Supplementary Materials

The following are available online at https://www.mdpi.com/2072-6694/12/6/1614/s1, Table S1: Number of metastatic lesions formed by A549 scr or A549 KOAGR2 cells in vivo, Table S2: Sequences of the primers used in quantitative PCR assays, Figure S1: AGR2 negatively correlates with ZEB1 in LUAD (lung adenocarcinoma). Oncomine box plots of AGR2 and ZEB1 levels in human lung adenocarcinoma and normal lung tissues in three various datasets (Okamaya, Selamat, Landi), Figure S2: Lung cancer patients’ mRNA expression profiling (*n* = 1439) to compare AGR2 vs. CDH1 and ZEB1 (http://ist.medisapiens.com), Figure S3: Graph for TCGA Lung adenocarcinoma data showing the gene expression of ZEB1 in lung adenocarcinoma patients with respect to AGR2 expression (http://xena.ucsc.edu), Figure S4: The AGR2 expression level in primary tumor site and metastasis. Oncomine box plots of AGR2 level in human lung adenocarcinoma in primary tumor site and in metastasis in two various datasets (Bittner, Bhattacharjee), Figure S5: Whole Western blots for Figure 2 showing all the bands, Figure S6: Whole Western blots for Figure 4A,B showing all the bands, Figure S7: Whole Western blots for Figure 4F,G showing all the bands, Figure S8: Whole Western blots for Figure 5 showing all the bands.
