# Supplementary material for: ZEB1/miR-200c/AGR2: A New Regulatory Loop Modulating the Epithelial-Mesenchymal Transition in Lung Adenocarcinomas
Source: Cancers (Basel). 2020 Jun 18;12(6):1614. doi: 10.3390/cancers12061614 (PMC7352583; doi:10.3390/cancers12061614)
Supplement: Supplementary file 1 [file cancers-12-01614-s001.pdf]

## Supplementary Materials

**Table S1.** Number of metastatic lesions formed by A549 scr or A549 KOAGR2 cells in vivo.

| Case | Number of Lung Metastasis Lesions (Diameter) |
|---|---|
| Mouse 1: A549 scr lung | 1 (0.2 cm) |
| Mouse 2: A549 scr lung | No metastasis lesions |
| Mouse 3: A549 scr lung | 1 (0.1 cm) |
| Mouse 4: A549 scr lung | No metastasis lesions |
| Mouse 5: A549 scr lung | No metastasis lesions |
| Mouse 1: A549 KOAGR2 lung | 9 (0.1–0.3 cm) |
| Mouse 2: A549 KOAGR2 lung | 6 (0.1–0.35 cm) |
| Mouse 3: A549 KOAGR2 lung | 1 (0.1 cm) |
| Mouse 4: A549 KOAGR2 lung | 4 (0.1–0.3 cm) |
| Mouse 5: A549 KOAGR2 lung | 7 (0.1–0.3 cm) |
| Mouse 6: A549 KOAGR2 lung | 6 (0.1–0.3 cm) |

**Table S2.** Sequences of the primers used in quantitative PCR assays.

| Gene | Forward | Reverse |
|---|---|---|
| *AGR2* | GGAGCTCTATATAAATCCAAGACAAGCA | GCCAATTTCTGGATTTCTTTATTTTC |
| *GAPDH* | GAAGGTGAAGGTCGGAGTC | GAAGATGGTGATGGGATTTC |
| *ZEB1* | CACTGGTGGTGGCCCATTAC | TGCACCATGCCCTGAGG |
| *AGR2 promotor* | TCGGGCCTCTGATTGCTA | AGTGTGACGTTCCCGAGT |

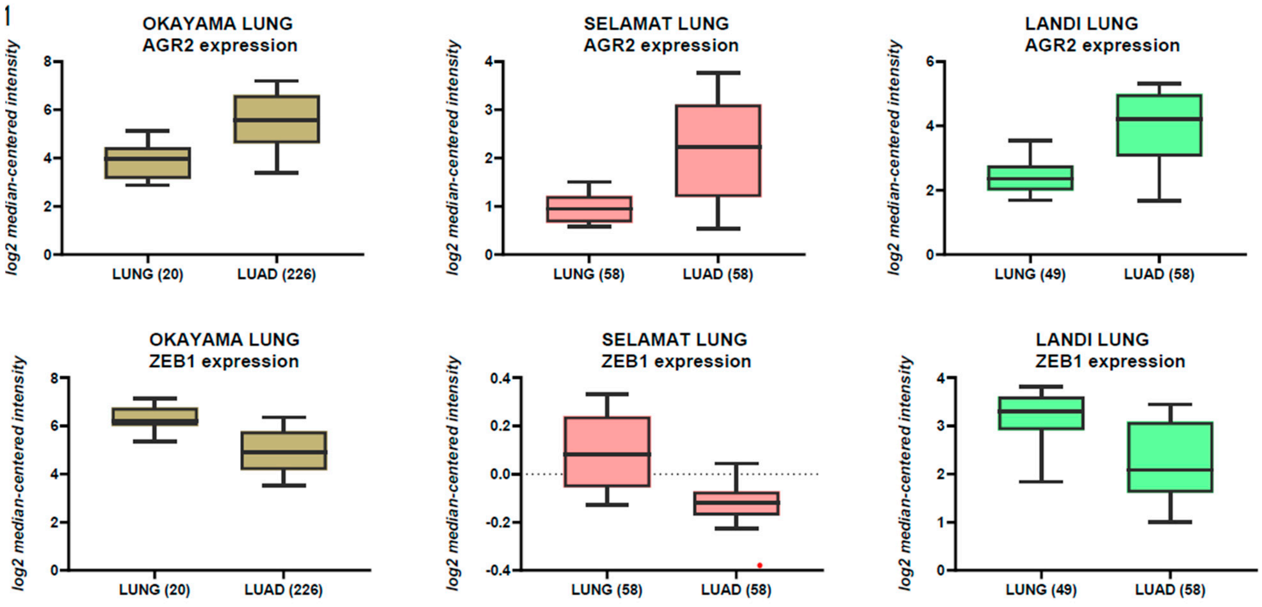


| Lung adenocarcinoma/normal lung log(fold changes) of *ZEB1* and *AGR2* genes in the Oncomine datasets. | | | | | |
|---|---|---|---|---|---|
| | DATASETS | | | | |
| Genes | Selamet | Hou | Landi | Su | Okayama |
| *ZEB1* | −1.15* | −1.14* | −1.804* | −2.814* | −2.350* |
| *AGR2* | 2.393* | 2.036* | 2.779* | 3.434* | 2.965* |

**Figure S1.** AGR2 negatively correlates with ZEB1 in LUAD (lung adenocarcinoma). Oncomine box plots of AGR2 and ZEB1 levels in human lung adenocarcinoma and normal lung tissues in three various datasets (Okamaya, Selamat, Landi). *$p < 0.05$

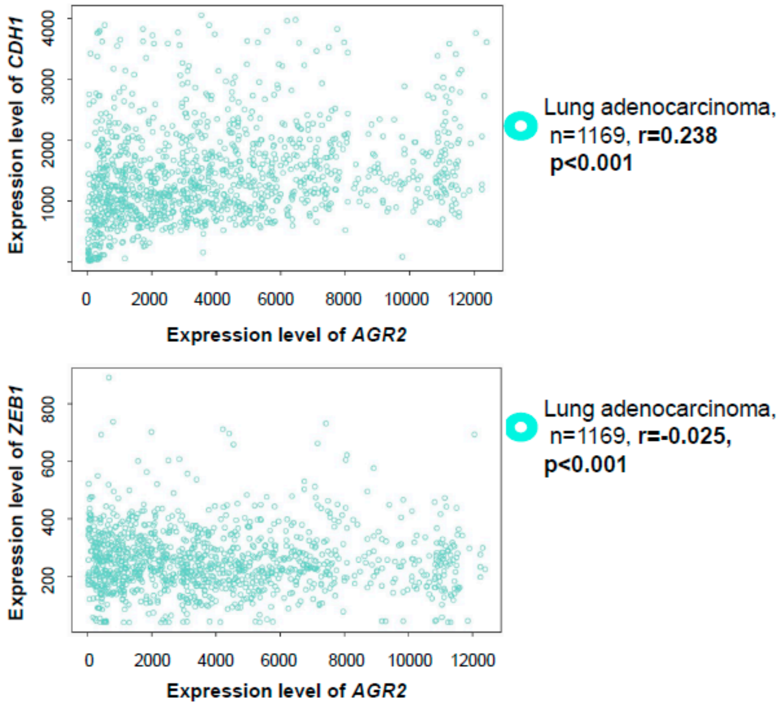


**Figure S2.** Lung cancer patients' mRNA expression profiling (*n* = 1439) to compare AGR2 vs. CDH1 and ZEB1 according to IST Online by Medisapiens Ltd.

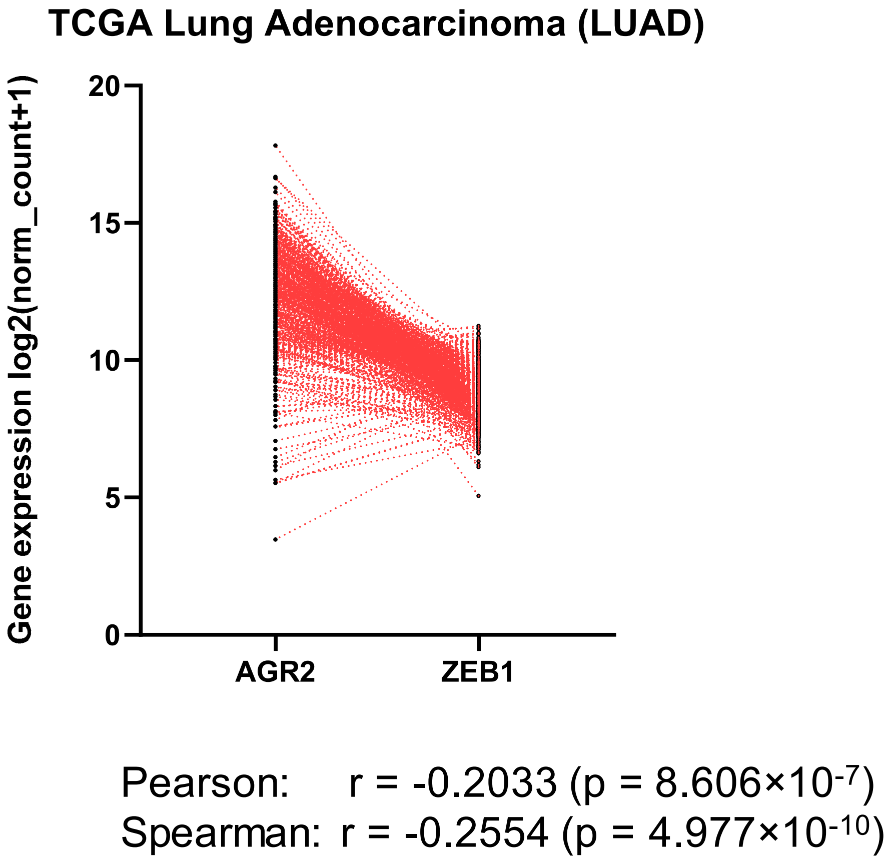


**Figure S3.** Graph for TCGA Lung adenocarcinoma data showing the gene expression of ZEB1 in lung adenocarcinoma patients with respect to AGR2 expression according to UCSC Xena.

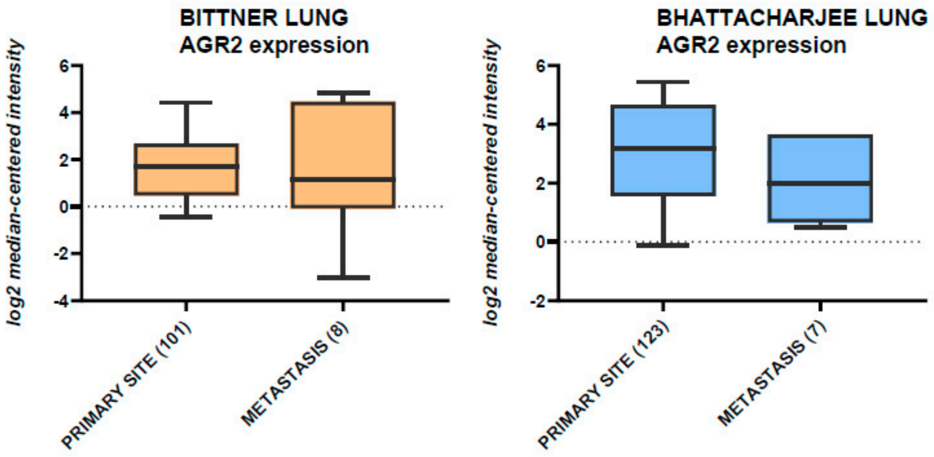


**Figure S4.** The AGR2 expression level in primary tumor site and metastasis. Oncomine box plots of AGR2 level in human lung adenocarcinoma in primary tumor site and in metastasis in two various datasets (Bittner, Bhattacharjee).

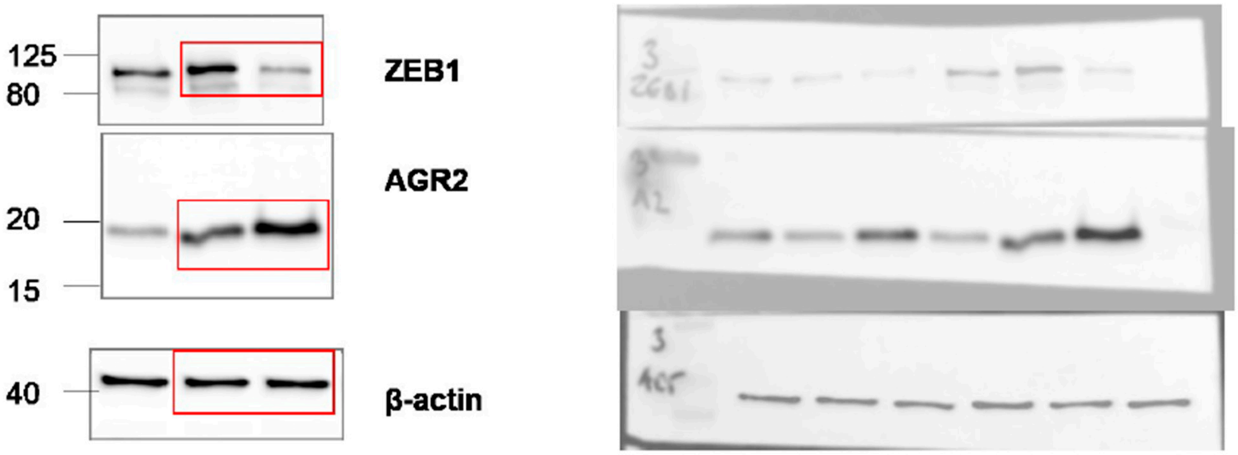


**Figure S5.** Whole Western blots for Figure 2 showing all the bands.

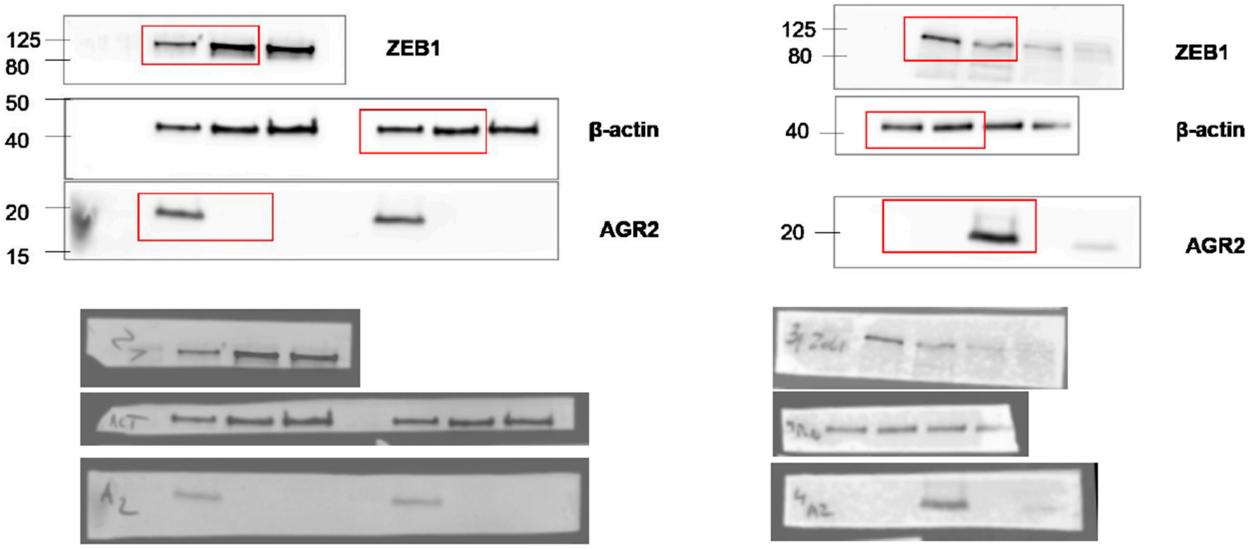


**Figure S6.** Whole Western blots for Figure 4A and 4B showing all the bands.

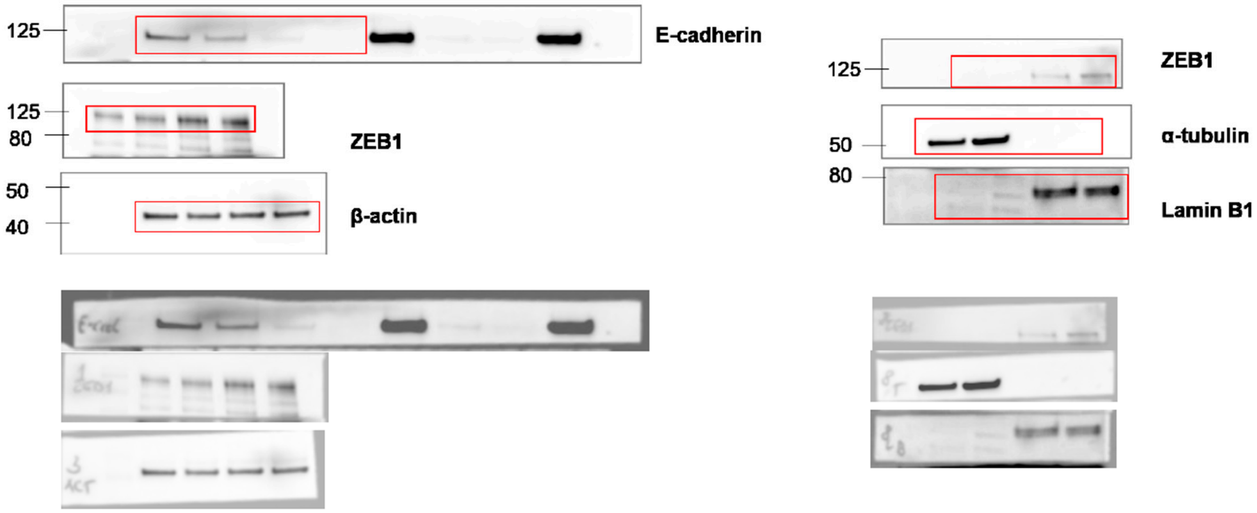


**Figure S7.** Whole Western blots for Figure 4F and 4G showing all the bands.

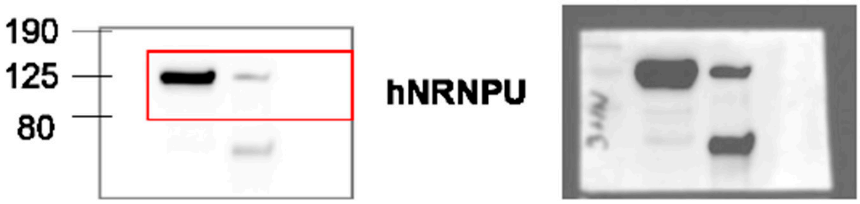


**Figure S8.** Whole Western blots for Figure 5 showing all the bands.
